# Exposure to wildfire-related PM_2.5_ and site-specific cancer mortality in Brazil from 2010 to 2016: A retrospective study

**DOI:** 10.1371/journal.pmed.1004103

**Published:** 2022-09-19

**Authors:** Pei Yu, Rongbin Xu, Shanshan Li, Xu Yue, Gongbo Chen, Tingting Ye, Micheline S. Z. S. Coêlho, Paulo H. N. Saldiva, Malcolm R. Sim, Michael J. Abramson, Yuming Guo

**Affiliations:** 1 School of Public Health and Preventive Medicine, Monash University, Melbourne, Victoria, Australia; 2 Jiangsu Key Laboratory of Atmospheric Environment Monitoring and Pollution Control, Collaborative Innovation Center of Atmospheric Environment and Equipment Technology, School of Environmental Sciences and Engineering, Nanjing University of Information Science & Technology, Nanjing, China; 3 Guangzhou Key Laboratory of Environmental Pollution and Health Risk Assessment, Guangdong Provincial Engineering Technology Research Center of Environmental Pollution and Health Risk Assessment, Department of Occupational and Environmental Health, School of Public Health, Sun Yat-sen University, Guangzhou, China; 4 Laboratory of Urban Health, Insper, São Paulo, Brazil; 5 Faculty of Medicine, University of São Paulo, São Paulo, Brazil; AUSTRALIA

## Abstract

**Background:**

Long-term exposure to fine particles ≤2.5 μm in diameter (PM_2.5_) has been linked to cancer mortality. However, the effect of wildfire-related PM_2.5_ exposure on cancer mortality risk is unknown. This study evaluates the association between wildfire-related PM_2.5_ and site-specific cancer mortality in Brazil, from 2010 to 2016.

**Methods and findings:**

Nationwide cancer death records were collected during 2010–2016 from the Brazilian Mortality Information System. Death records were linked with municipal-level wildfire- and non-wildfire-related PM_2.5_ concentrations, at a resolution of 2.0° latitude by 2.5° longitude. We applied a variant difference-in-differences approach with quasi-Poisson regression, adjusting for seasonal temperature and gross domestic product (GDP) per capita. Relative risks (RRs) and 95% confidence intervals (CIs) for the exposure for specific cancer sites were estimated. Attributable fractions and cancer deaths were also calculated. In total, 1,332,526 adult cancer deaths (age ≥ 20 years), from 5,565 Brazilian municipalities, covering 136 million adults were included. The mean annual wildfire-related PM_2.5_ concentration was 2.38 μg/m^3^, and the annual non-wildfire-related PM_2.5_ concentration was 8.20 μg/m^3^. The RR for mortality from all cancers was 1.02 (95% CI 1.01–1.03, *p* < 0.001) per 1-μg/m^3^ increase of wildfire-related PM_2.5_ concentration, which was higher than the RR per 1-μg/m^3^ increase of non-wildfire-related PM_2.5_ (1.01 [95% CI 1.00–1.01], *p =* 0.007, with *p* for difference = 0.003). Wildfire-related PM_2.5_ was associated with mortality from cancers of the nasopharynx (1.10 [95% CI 1.04–1.16], *p =* 0.002), esophagus (1.05 [95% CI 1.01–1.08], *p =* 0.012), stomach (1.03 [95% CI 1.01–1.06], *p =* 0.017), colon/rectum (1.08 [95% CI 1.05–1.11], *p <* 0.001), larynx (1.06 [95% CI 1.02–1.11], *p =* 0.003), skin (1.06 [95% CI 1.00–1.12], *p =* 0.003), breast (1.04 [95% CI 1.01–1.06], *p =* 0.007), prostate (1.03 [95% CI 1.01–1.06], *p =* 0.019), and testis (1.10 [95% CI 1.03–1.17], *p =* 0.002). For all cancers combined, the attributable deaths were 37 per 100,000 population and ranged from 18/100,000 in the Northeast Region of Brazil to 71/100,000 in the Central-West Region. Study limitations included a potential lack of assessment of the joint effects of gaseous pollutants, an inability to capture the migration of residents, and an inability to adjust for some potential confounders.

**Conclusions:**

Exposure to wildfire-related PM_2.5_ can increase the risks of cancer mortality for many cancer sites, and the effect for wildfire-related PM_2.5_ was higher than for PM_2.5_ from non-wildfire sources.

## Abstract 2

Pei Yu and colleagues explore the association between exposure to wildfire related PM2.5 and site-specific cancer mortality in Brazil from 2010 to 2016.

## Author summary

**Why was this study done?:**

- Cancer is a leading cause of death worldwide, and cancer-related deaths are projected to increase in the future in all countries, including Brazil.
- Given the increasing frequency and duration of wildfires in recent decades, the effects of wildfires on health need to be better understood.
- The association between wildfire PM_2.5_ and site-specific cancer mortality remains unclear.

**What did the researchers do and find?:**

- We conducted a retrospective study using data from the Mortality Information System in Brazil to assess whether wildfire-related PM_2.5_ exposure was associated with mortality from cancer for common cancer sites in adults.
- Municipality-level wildfire- and non-wildfire-related PM_2.5_ concentrations were estimated and linked with the mortality data.
- We found that wildfire-related PM_2.5_ exposure was associated with cancer mortality for various common cancer sites in adults in Brazil, and higher effects were observed for wildfire-related PM_2.5_ than for non-wildfire sources of PM_2.5_.

**What do these findings mean?:**

- Our findings suggest a high wildfire-related PM_2.5_ attributable cancer burden, among adults in Brazil.
- The potentially higher risk of wildfire-related PM_2.5_ compared with non-wildfire-related PM_2.5_ for all cancers combined suggests that the wildfire control and systemic prevention strategies are warranted, to reduce cancer mortality risk in Brazil. This could be a health co-benefit of measures to preserve the Amazon rainforest and limit climate change.

## Introduction

Wildfires have become more frequent under climate change in recent years and pose a serious threat to human health. Even those living many kilometers away from wildfires are exposed to their smoke; thus, the health impacts of wildfires on the general population are a concern. Wildfires emit high concentrations of air pollutants and hazardous substances, including fine particles ≤2.5 μm in diameter (PM_2.5_), which are regarded as the fire tracer in epidemiological studies.

It has been estimated that 0.62% of all-cause deaths are annually attributable to the acute impacts of wildfire-related PM_2.5_ exposure globally [1]. Apart from death, increased risks of morbidity from respiratory diseases, cardiovascular diseases, low birth weight and preterm birth, and influenza were observed after acute short-term wildfire smoke exposure [2]. Only a few studies have reported the long-term effect of wildfire-related PM_2.5_ (e.g., on general health and lung capacity) [3–6]. Currently, there are research gaps regarding the potential health impacts of long-term wildfire smoke exposure, including on the risk of cancer.

Occupational studies investigating the risk of cancer in firefighters found that firefighters who experienced a high degree of wildfire smoke exposure had higher risks of cancer compared to firefighters exposed to limited wildfire smoke [7–9]. Wildfire-related particles were suggested to have smaller sizes and to contain more oxidative and proinflammatory components than urban sources of particles [2,10]. Thus, wildfire-related PM_2.5_ exposure could also increase cancer mortality in the general population, and the effect may be higher than for non-wildfire PM_2.5_ sources.

The majority of the Amazon rainforest, which represents over half of the planet’s rainforests, is contained within Brazil [11]. The current unprecedented scale of wildfires means many Brazilian people are exposed to fire smoke. Given that toxic smoke from wildfires travels long distances with wind, the assessment of the health effects of wildfires should not be limited to firefighters. If the association between wildfire-related PM_2.5_ and cancer mortality is higher than that for non-wildfire PM_2.5_, cancer could be an important consideration when making public health allocation strategies, especially in Brazil. Assessment of the impact of exposure to wildfire-related PM_2.5_ upon mortality from all types of cancer would also inform public health measures to improve cancer survival.

To address this important issue, in this study, we analyze the associations between wildfire-related PM_2.5_ and cancer-specific mortality, using national mortality data spanning 2010–2016 in Brazil. This study also compares the impacts of non-wildfire-related and wildfire-related PM_2.5_ on cancer mortality. Finally, we estimate regional cancer death counts attributable to wildfire-related PM_2.5_.

## Methods

This study is reported as per the REporting of studies Conducted using Observational Routinely-collected Data (RECORD) statement (S1 RECORD Checklist).

### Protocol

This research was conducted using data from the Brazil Mortality Information System. Our study did not employ a prospective protocol. Analyses were first planned and performed in August 2021. During peer review, we added a figure of sensitivity analysis and a table comparing the effect on cancer mortality and drowning (as the negative control). Changes to the paper were also made at the request of peer reviewers.

### Study population

Individual death records from 1 January 2010 to 31 December 2016 were collected from the Brazil Mortality Information System (Sistema de Informação sobre Mortalidade) [12]. Complete records from 5,565 municipalities, covering about 99.98% of the Brazilian population distributed in the 5 regions of Brazil, were included in the analyses. Municipalities with missing mortality data and records with missing age or sex were excluded from the analyses. Each death record included information on municipality, age, sex, date, and primary cause of death, coded according to the International Statistical Classification of Diseases and Related Health Problems—10th Revision (ICD-10, https://icd.who.int/browse10/2019/en). Cancer deaths were totaled for every municipality-year and grouped as follows: oral (C00–C10, C12–C14), nasopharynx (C11), esophagus (C15), stomach (C16), colon/rectum (C18–C21), liver (C22), gallbladder (C23–C24), pancreas (C25), larynx (C32), lung (C33–C34), bone (C40–C41), skin (C43), breast (C50), cervix (C53), uterus (C54–C55), ovary (C56), prostate (C61), testis (C62), kidney (C64–C66), bladder (C67), brain (C70–C72), lymphoma (C81–C85), and leukemia (C91–C95). The death counts were also divided by sex and age groups (male versus female; aged 20–59 versus 60+ years). Child and adolescent cancers are not the same as adult cancers, with different types, treatment, and survival [13,14]; thus, only cancer deaths in individuals aged ≥20 years were included in the analyses.

### Pollution exposure

Daily all-source PM_2.5_ and wildfire-related PM_2.5_ were estimated during the study period; the details of model development and validation have been described in our previous work [1,15,16]. In summary, fire-induced change in PM_2.5_ was predicted by the chemical transport model GEOS-Chem (version 12.0.0) as the difference in PM_2.5_ from simulations with and without fire emissions. The anthropogenic emissions from 5 fire sources (boreal forest fires; tropical forest fires; savanna, grassland, and shrubland fires; temperate forest fires; agricultural waste burning) were from the EDGAR v4.2 inventory (http://edgar.jrc.ec.europa.eu/). The all-source PM_2.5_ was then downscaled from the original resolution of 2.0° latitude × 2.5° longitude to a higher resolution of 0.25° × 0.25° using a random forest model, taking into account the impacts of meteorology on PM_2.5_ in the fine grid cells. The downscaled all-source PM_2.5_ from GEOS-Chem was validated against ground-level PM_2.5_ monitored at 6,882 global sites, with a high coefficient of determination of up to 0.865 [1]. Then wildfire-related PM_2.5_ was derived as the product of all-source PM_2.5_ and the wildfire-to-all ratio calculated by the GEOS-Chem model. Annual mean non-wildfire- and wildfire-related PM_2.5_ were calculated from daily non-wildfire PM_2.5_ and wildfire-related PM_2.5_ during 2000–2016. The official geographical boundaries of municipalities were downloaded from the website of the Brazilian Institute of Geography and Statistics (BIGS; https://www.ibge.gov.br/pt/inicio.html).

### Other covariates

Daily mean temperatures were calculated from hourly temperature records from the European Centre for Medium-Range Weather Forecasts Reanalysis v5 (ERA5) dataset, with a 0.25° × 0.25° (approximately 28 km × 28 km) spatial resolution. This dataset has global coverage and is comparable to weather station observations in evaluating temperature–mortality associations [17]. The municipality-level temperature was represented by the temperature of the grid at the geographical center of each municipality.

Municipality-level population size and gross domestic product (GDP) per capita for every year during the study period were downloaded from BIGS and then adjusted to United States dollars at the current price, according to the consumer price index during 2008–2020 and the average exchange rate in 2020 [18,19]. All variables were linked to death cases according to municipality and year.

### Statistical analysis: PM_2.5_–cancer mortality association

A variant difference-in-differences (DID) approach with quasi-Poisson regression was applied to examine the associations between exposure and all cancers and site-specific cancer mortality. The essence of the variant DID design is that the difference in temporal concentrations (wildfire- and non-wildfire-related PM_2.5_ in this study) is related to the difference in cancer mortality rates in each location during the study period [20]. Factors that keep stable during the study time and time trends in confounders that changed similarly across locations are controlled. Confounders that correlate with the wildfire- or non-wildfire-related PM_2.5_ concentrations and that change differently across regions by time should be adjusted in the model. The parameters of the variables are defined based on previous studies [21–23]. Temperature has been demonstrated to be associated with cancer mortality and thus is fitted in the main model [24,25]. Socioeconomic factors are represented by GDP per capita. Cancer-specific mortality associations were evaluated using the following model:

$$\ln\left[E(Y_{s,t})\right]=\beta_{0}{+\beta_{1}I_{s}+\beta_{2}I_{t}+\beta_{3}PM}_{2.5s,t}+{ln(Pop}_{s,t})+\beta_{4}{Temp}_{summers,t}+\beta_{5}{Temp}_{winters,t}+{\beta_{6}SD(Temp}_{summers,t})+\beta_{7}{SD(Temp}_{winters,t})+\beta_{8}{GDP\_per\_capita}_{s,t}$$

where *Y*_*s*,*t*_ represents the number of cases in municipality *s*, year *t*; *I*_*s*_ is a dummy variable for municipality *s*; *I*_*t*_ is a dummy variable for year *t*; PM_2.5*s*,*t*_ is the average wildfire- or non-wildfire-related PM_2.5_ in municipality *s*, year *t*; β_*s*_ is the intercept or slope for the linear terms; ln(Pop_*s*,*t*_) is an offset term representing the natural log of the population in municipality *s*, year *t*; and Temp values are the means of summer and winter temperatures and their standard deviations (SDs).

We also performed subgroup analyses by age group (20–59 years versus 60 years or above) and sex. We used fixed-effects meta-analyses to compare the effect estimates between sex and age groups. All results are expressed as relative risks (RRs) and 95% confidence intervals (95% CIs) per 1-μg/m^3^ increase in annual average PM_2.5_ concentration. Several sensitivity analyses were performed—adding gas pollutants (CO, NO_2_, O_3_, SO_2_), Normalized Difference Vegetation Index (NDVI), and nighttime light (NTL); modeling the summer and winter temperatures using natural cubic splines with 2 or 3 degrees of freedom; and removing GDP per capita from the main model—to check the robustness of the main findings.

R software (version 3.4.3; https://www.r-project.org/) was used to perform all data analyses. The “gnm” package was used to perform the conditional Poisson regression model. The “mvmeta” package was used to compare the subgroup differences. Statistical significance was defined as a 2-sided *p-*value < 0.05.

This study was approved by the Monash University Human Research Ethics Committee. The Brazilian Ministry of Health did not require ethics approval or informed consent for secondary analysis of aggregated anonymized data from the Mortality Information System.

## Results

**Fig 1:**
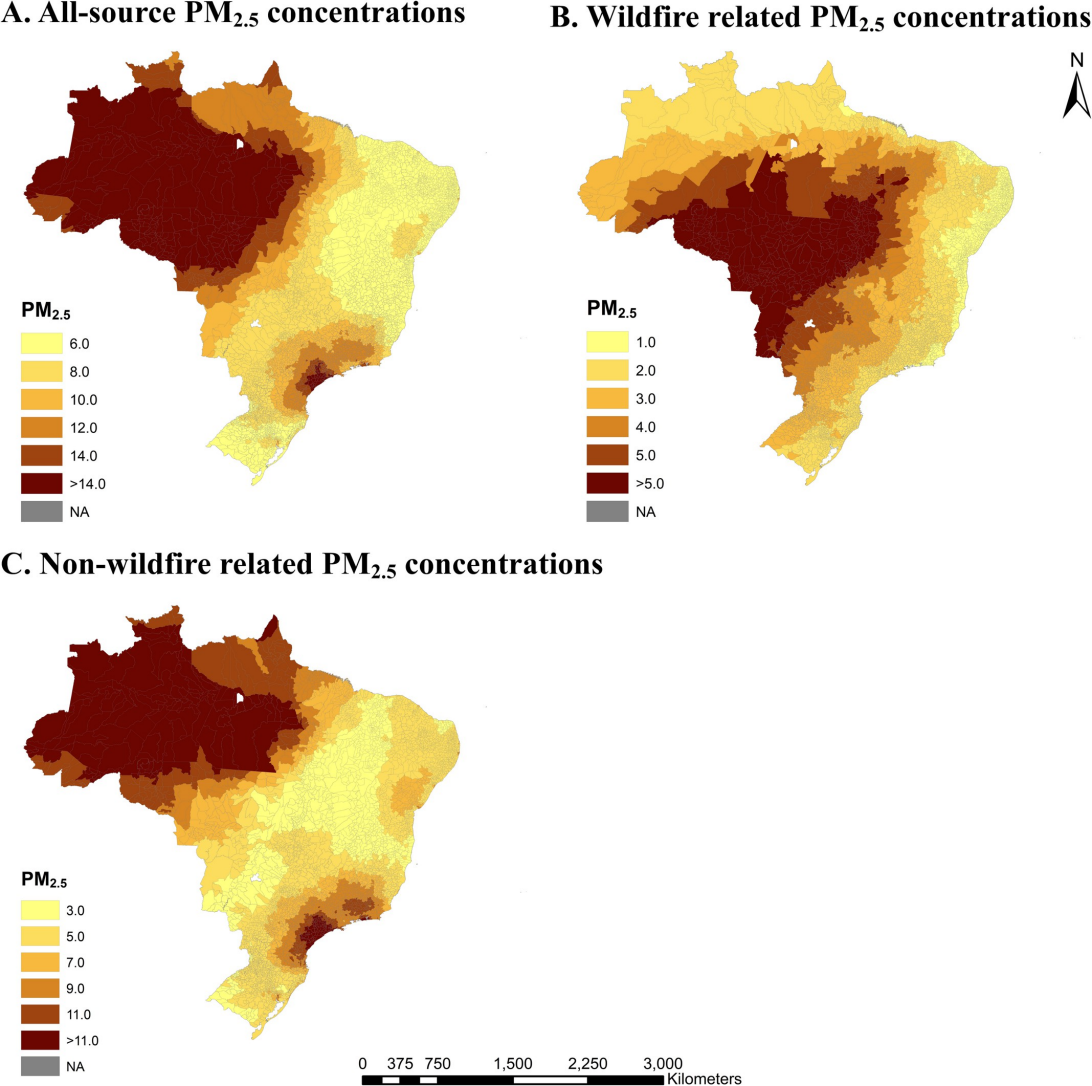
Estimated annual all-source PM_2.5_ concentrations, wildfire-related PM_2.5_ concentrations, and non-wildfire-related PM_2.5_ concentrations for municipalities in Brazil from 2010−2016. (A) All-source PM_2.5_ concentrations; (B) wildfire-related PM_2.5_ concentrations; (C) non-wildfire-related PM_2.5_ concentrations. NA, not available. The base map of this figure was downloaded from the Brazilian Institute of Geography and Statistics (https://www.ibge.gov.br/en/geosciences/territorial-organization/territorial-meshes/18890-municipal-mesh.html?edicao=30154&t=downloads); the base map was free and open-access.

A total of 1,332,526 adult cancer death records from 5,565 municipalities, with municipality areas ranging from 3.56 to 159,533 km^2^, covering almost the total population of Brazil from 2010 to 2016 were included in the main analyses. Cancer death counts from common cancer sites are presented in S1 Table. Of all records included, death counts varied from 0 to 123,571 within municipalities. Mean annual wildfire-related PM_2.5_ was 2.38 μg/m^3^ (ranging from 0.60 to 12.49 μg/m^3^), with regional variability (Table 1). The distribution of wildfire-related PM_2.5_ showed a radial pattern from municipalities in the Central-West Region and surrounding areas (Fig 1). The proportion of wildfire-related PM_2.5_ of all-source PM_2.5_ is shown in S1 Fig. High total PM_2.5_ concentration in the North Region was observed, which may be associated with volcanic SO_2_, lightning NOx, biogenic soil NO, ocean emissions, biogenic emissions, very short-lived iodine and bromine species, and decaying plants (S2 Fig).

**Table 1:** Descriptive characteristics of study participants and summary statistics for the 5,565 municipalities in Brazil.

Characteristic	Number of participants	Mean	SD	Median	Minimum	Maximum
**Health data**						
Cancer deaths (persons)	1,332,526	239	2,350	29	0	123,571
Age (years)						
20–59	420,792	7	1,586	76	0	39,375
≥60	911,734	164	767	22	0	84,196
Sex (persons)						
Males	709,535	128	1,163	17	0	61,533
Females	622,887	112	1,189	12	0	62,038
**Demographic data**						
Population size (persons)	199,997,499	35,938	212,436	11,306	0	11,779,640
Adult population size (persons)	136,303,472	24,493	152,255	7,490	0	8,457,673
Age (years)						
20–59	112,977,597	20,301	124,834	6,097	460	6,932,700
≥60	23,325,874	4,192	27,744	1,392	89	1,524,974
Sex (persons)						
Males	65,496,608	11,769	70,120	3,816	306	3,902,467
Females	70,806,864	12,724	82,151	3,704	274	4,555,206
**Environmental data**						
Wildfire PM_2.5_ (μg/m^3^)	—	2.38	1.62	1.94	0.60	12.49
lag1^a^	—	2.26	1.48	1.85	0.58	11.08
lag0–1^b^	—	2.32	1.55	1.89	0.59	11.79
Non-wildfire PM_2.5_ (μg/m^3^)	—	8.20	1.50	7.89	4.16	17.11
lag1^a^	—	8.22	1.48	7.92	4.16	16.86
lag0–1^b^	—					
Mean summer temperature (°C)	—	25.27	1.86	25.47	18.23	29.92
SD of summer temperature (°C)	—	1.45	0.45	1.48	0.41	2.63
Mean winter temperature (°C)	—	21.33	4.47	21.64	10.55	30.28
SD of winter temperature (°C)	—	1.96	1.21	1.57	0.37	4.85
**Socioeconomic data**						
GDP per capita (USD)	—	4,333	4,636.40	3,249	807	146,701

^a^lag1 refers to 1 year prior to the current year.

^b^lag0–1 refers to 2-year average (current year and 1 year prior to the current year) concentration.

GDP, gross domestic product; SD, standard deviation; USD, US dollars.

**Fig 2:**
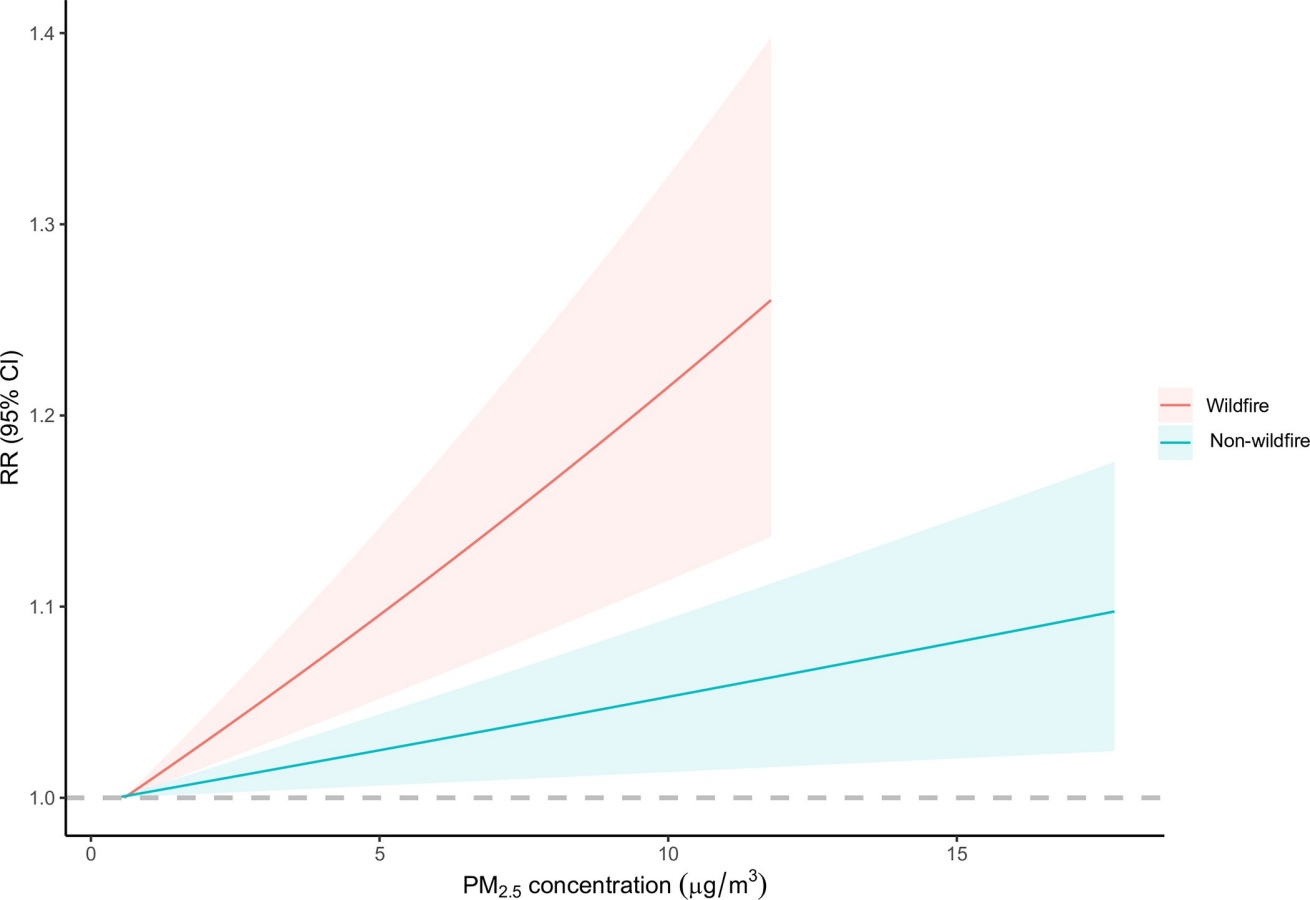
Estimated RR (95% CI) for the association between a 1-μg/m^3^ increase in lag0–1-year wildfire-related PM_2.5_, and non-wildfire-related PM_2.5_ and cancer mortality for 2010–2016. The solid lines represent the RR, and the shaded areas represent the 95% CI. The model, by its design, controlled for factors that were stable across the study period or had similar trend across geographical locations, and also adjusted for spatial-temporal factors including seasonal temperature and GDP per capita. CI, confidence interval; RR, relative risk.

The associations between a 1-μg/m^3^ increase of wildfire-related PM_2.5_ concentration and cancer mortality risks for single lag years and moving average lag years are shown in S3 Fig. Significant associations were observed in the current year and 1 year before the death for all cancers combined. Thus 2-year moving average concentration was used in later analyses. The relationships between wildfire-related PM_2.5_ and total cancer mortality modeled by natural cubic splines with 1–4 degrees of freedom were similar, and linear analysis had the lowest QBIC (Quasi-Bayesian Information Criteria), indicating linear associations between wildfire-related PM_2.5_ and total cancer mortality (S4 Fig). Compared with non-wildfire PM_2.5_, people were more vulnerable to wildfire-related PM_2.5_ (Fig 2).

**Fig 3:**
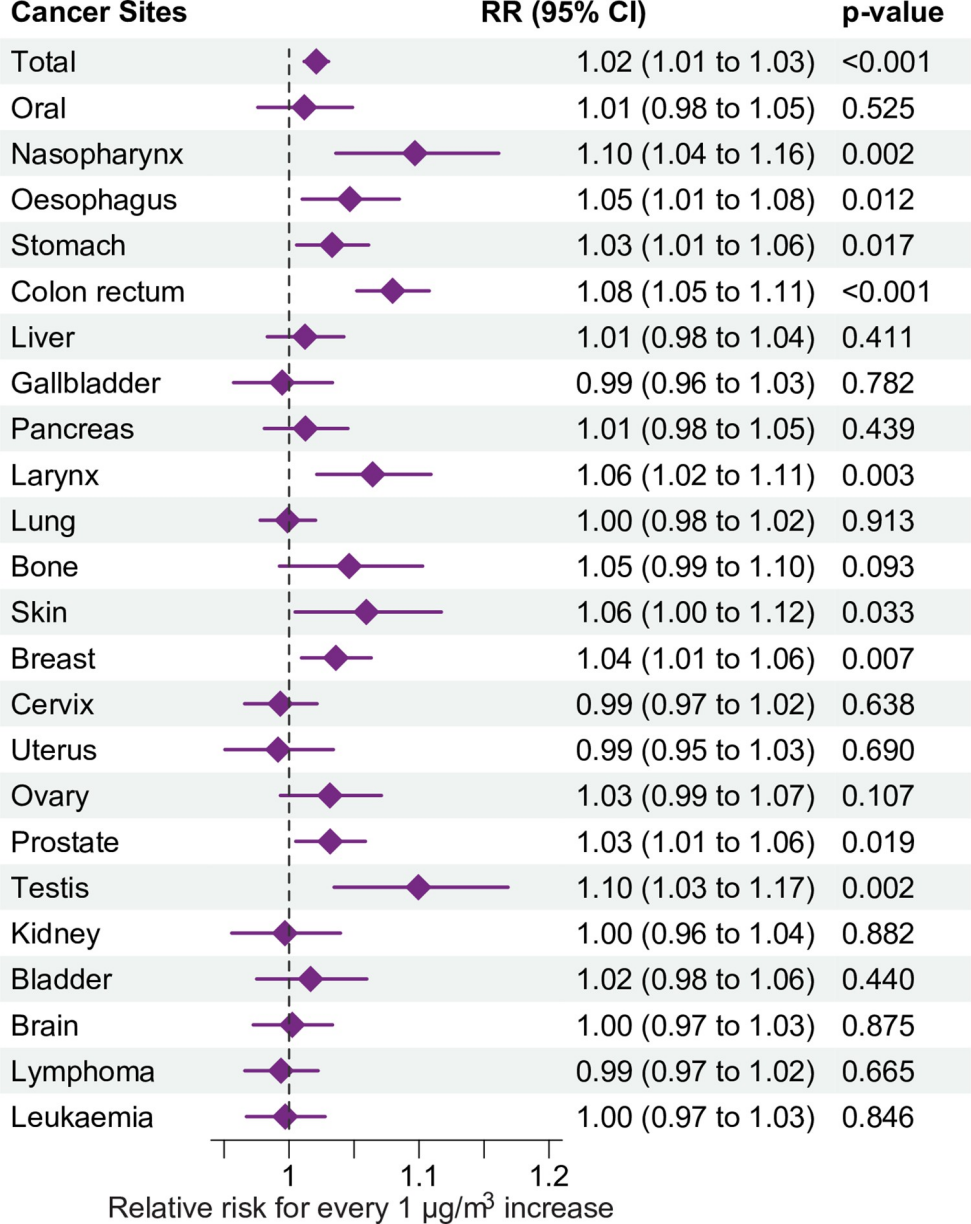
Estimated RRs and 95% CIs for the association between a 1-μg/m^3^ increase in 2-year average (lag0–1) wildfire-related PM_2.5_ and all-cancer and site-specific cancer mortality, from 2010–2016. The vertical dashed line represents the reference line for RR = 1, helping to compare the effect estimates with the null hypothesis; the error bars represent 95% CIs. The model, by its design, controlled for factors that were stable across the study period or had similar trend across geographical locations, and also adjusted for spatial-temporal factors including seasonal temperature and GDP per capita. CI, confidence interval; RR, relative risk.

The RR for mortality for all cancers combined per 1-μg/m^3^ increase of wildfire-related PM_2.5_ concentration was 1.02 (95% CI 1.01–1.03, *p <* 0.001). Cancer mortality was higher for wildfire-related PM_2.5_ than for other sources of PM_2.5_ (1.01 [95% CI 1.00–1.01], *p =* 0.007, *p* for difference = 0.003). Wildfire-related PM_2.5_ was associated with mortality from cancers of the nasopharynx (1.10 [95% CI 1.04–1.16], *p =* 0.002), esophagus (1.05 [95% CI 1.01–1.08], *p =* 0.012), stomach (1.03 [95% CI 1.01–1.06], *p =* 0.017), colon/rectum (1.08 [95% CI 1.05–1.11], *p <* 0.001), larynx (1.06 [95% CI 1.02–1.11], *p =* 0.003), breast (1.04 [95% CI 1.01–1.06], *p =* 0.007), prostate (1.03 [95% CI 1.01–1.06], *p =* 0.019), and testis (1.10 [95% CI 1.03–1.17], *p =* 0.002) (Fig 3). However, no significant association with lung cancer mortality was observed (1.00 [95% CI 0.98–1.01], *p =* 0.503). RRs associated with wildfire-related PM_2.5_ were greater than RRs for non-wildfire PM_2.5_ for colorectal (1.03 [95% CI 1.02–1.04], *p =* 0.001) and testis (1.10 [95% CI 1.03–1.17], *p <* 0.001) cancer mortality. Though no significant association was observed between cervical cancer and wildfire-related PM_2.5_, adverse effects (1.03 [95% CI 1.01–1.04], *p =* 0.001) were found for non-wildfire PM_2.5_ (S5 Fig).

**Fig 4:**
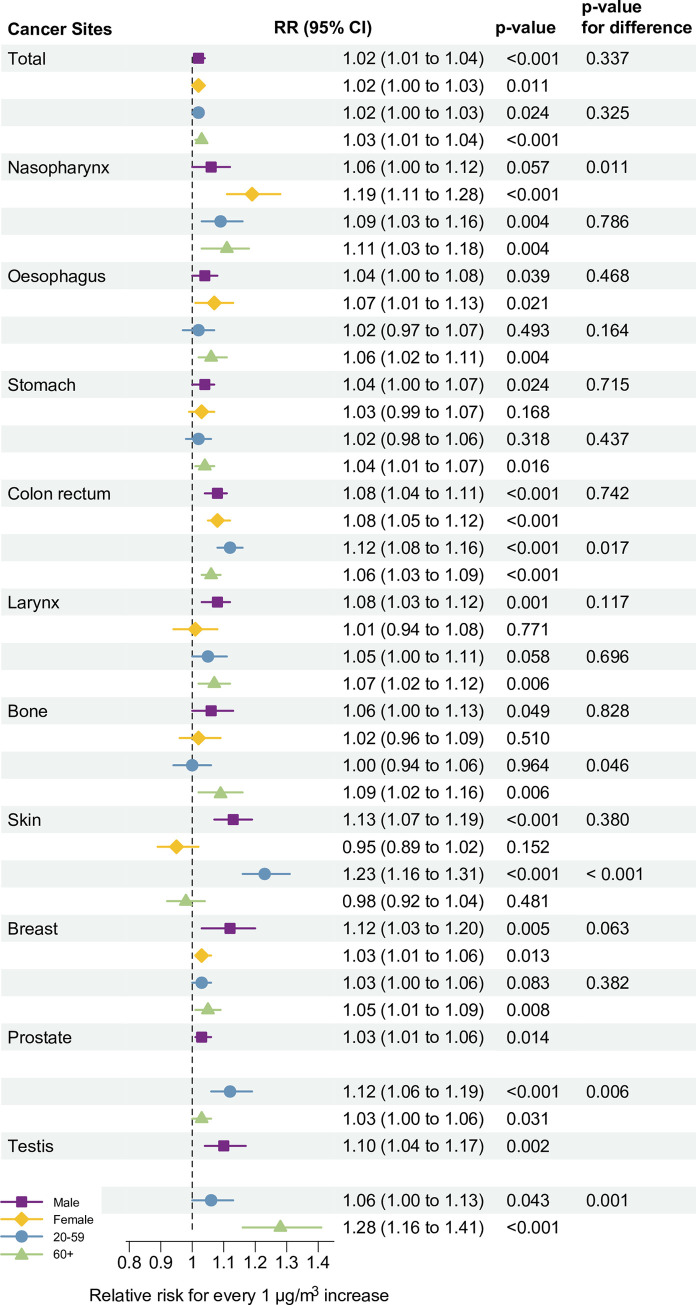
Estimated RRs (95% CIs) for the association between a 1-μg/m^3^ increase in 2-year average (lag0–1) wildfire-related PM_2.5_ and cancer mortality, from 2010–2016, by sex and age. The vertical dashed line represents the reference line for RR = 1, helping to compare the effect estimates with the null hypothesis; the error bars represent 95% CIs. The model, by its design, controlled for factors that were stable across the study period or had similar trend across geographical locations, and also adjusted for spatial-temporal factors including seasonal temperature and GDP per capita. The *p*-values for differences were estimated by fixed-effects meta-analysis with no statistical adjustment, because models were based on the same sample. CI, confidence interval; RR, relative risk.

To further examine vulnerable cancer sites and population subgroups, stratified analyses for mortality from potentially affected cancers by age and sex are shown in Fig 4. There was no significant difference for all cancers combined between males (1.02 [95% CI 1.01–1.04], *p <* 0.001) and females (1.02 [95% CI 1.00–1.03], *p =* 0.011; *p* for difference = 0.337) or between people aged 20–59 years (1.02 [95% CI 1.00–1.03], *p =* 0.024) and people 60 years or older (1.03 [95% CI 1.01–1.04], *p <* 0.001; *p* for difference = 0.325). The RR for mortality from skin cancers was more pronounced in males, while a higher RR for nasopharyngeal cancer mortality was observed in females. Notably, higher risks were observed for cancers of the colon/rectum, skin, and prostate among people aged 20–59 years, and higher RR for testicular cancer mortality was observed among people aged 60 years and older (Fig 4). Subgroup analyses for other cancer sites are shown in S6 Fig. Lung cancer mortality was significantly associated with non-wildfire PM_2.5_ in females (1.02 [95% CI 1.00–1.03], *p =* 0.013) and people 60 years or older (1.01 [95% CI 1.00–1.02], *p =* 0.010) (S7 Fig).

**Fig 5:**
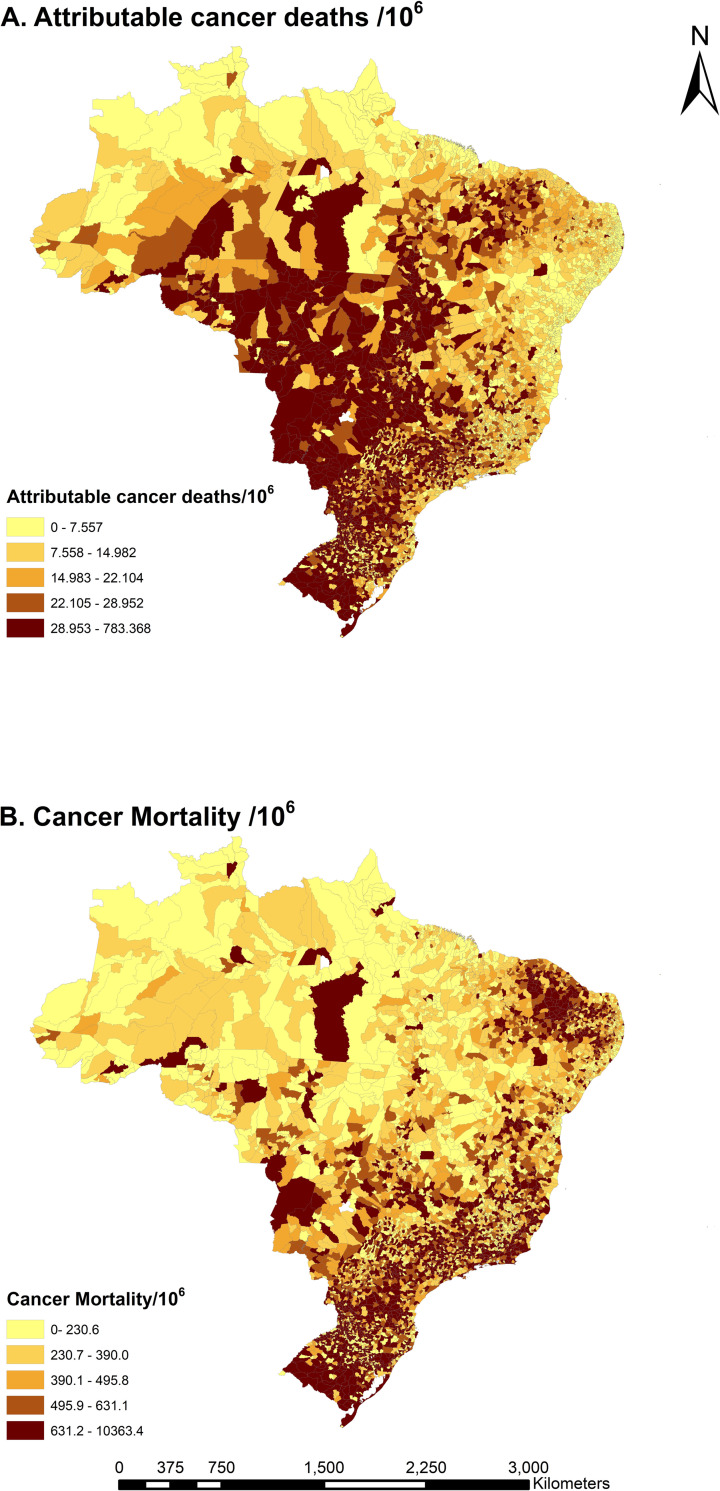
Total attributable cancer deaths and cancer mortality per 100,000 population for municipalities in Brazil from 2010–2016. (A) Attributable cancer deaths and (B) cancer mortality per 100,000 population. The base map of this figure was downloaded from the Brazilian Institute of Geography and Statistics (https://www.ibge.gov.br/en/geosciences/territorial-organization/territorial-meshes/18890-municipal-mesh.html?edicao=30154&t=downloads); the base map was free and open-access.

Wildfire-related PM_2.5_ attributable cancer deaths ranged from 0 to 822/100,000 population for municipalities during the study period, assuming the association is causal (Fig 5). In total, there were 53,135 cancer deaths (95% CI 30,743–75,322) attributable to 2-year average wildfire-related PM_2.5_ exposure from 2010 to 2016. Though the highest cancer mortality rate was in the South Region, the number of attributable cancer deaths per 100,000 population was higher in the Central-West Region (75/100,000). Males and people 60 years or older experienced a higher cancer burden. Along with increased cancer cases and higher wildfire-related PM_2.5_ exposure, the number of attributable cancer deaths was more pronounced in 2015 and 2016, which is consistent with the high wildfire-related PM_2.5_ exposure in those years (Table 2). State-level attributable cancer deaths are presented in S2 Table.

**Table 2:** Cancer deaths and attributable cancer deaths associated with 2-year average wildfire-related PM_2.5_ exposure by region, age, sex, and year during 2010–2016.

Factor	Number of cancer deaths	Cancer mortality/10^6^	Attributable cancer deaths 95% CI	Attributable cancer deaths/10^6^ 95% CI
Total	1,332,526	977.62	50,621 (28,212−72,822)	37.14 (20.70−53.43)
Region				
Central-West	83,147	819.13	7,229 (4,029–10,399)	71.21 (39.69–102.45)
Northeast	281,089	781.27	6,566 (3,659–9,446)	18.25 (10.17–26.25)
North	60,475	596.03	3,743 (2,086–5,384)	36.89 (20.56–53.06)
Southeast	646,683	1,082.00	22,725 (12,665–32,692)	38.02 (21.19–54.7)
South	261,132	1,288.86	10,358 (5,773–14,901)	51.12 (28.49–73.55)
Age (years)				
20–59	420,792	53.21	12,381 (1,635–22,978)	10.96 (1.45–20.34)
60+	911,734	558.38	41,350 (23,198–29,304)	177.27 (99.45–254.24)
Sex				
Males	709,535	154.76	32,549 (16,968–47,946)	49.70 (25.91–73.20)
Females	622,887	125.67	18,074 (4,210–31,767)	25.53 (2.95–44.86)
Year				
2010	172,715	133.32	6,361 (3,545–9,150)	4.91 (2.74–7.06)
2011	177,971	134.98	6,902 (3,847–9,930)	5.23 (2.92–7.53)
2012	184,680	137.71	6,467 (3,604–9,303)	4.82 (2.69–6.94)
2013	190,192	139.53	6,491 (3,617–9,338)	4.76 (2.65–6.85)
2014	195,432	141.07	6,906 (3,849–9,935)	4.98 (2.78–7.17)
2015	203,071	144.26	8,785 (4,896–12,638)	6.24 (3.48–8.98)
2016	208,465	145.77	8,709 (4,854–12,529)	6.09 (3.39–8.76)

Sensitivity analyses showed that our estimation was robust. The estimations using 2-year average (lag0–1) and three-year average (lag0–2) wildfire-related PM_2.5_ concentrations were similar (S3 Fig). We also compared associations estimated by adding other air pollutants (CO, NO_2_, O_3_, SO_2_), NDVI, and nighttime light; changing the degrees of freedom of temperature; and removing GDP per capita from the model (S3 Table). Unintentional drowning was used as the outcome for a negative control analysis: Drowning was not significantly associated with wildfire-related PM_2.5_ exposure (S4 Table).

## Discussion

We did a national analysis of the association between wildfire-related PM_2.5_ exposure and cancer mortality. We found that wildfire-related PM_2.5_ was significantly associated with an increased risk of all-cause cancer death in Brazil during 2010–2016. Increased risks were detected for cancers of the nasopharynx, esophagus, stomach, colon/rectum, larynx, skin, breast, prostate, and testis. Notably, we found that people may be more vulnerable to wildfire smoke than non-wildfire PM_2.5_ sources, especially for esophageal, colorectal, and testicular cancer. To our best knowledge, this study is the first to specifically focus on associations between wildfire-related PM_2.5_ and site-specific cancer mortality. The disease burden attributable to wildfire may be higher than previous estimates based on respiratory and cardiovascular diseases.

Cancer mortality is an outcome reflecting both the incidence of cancer and survival after diagnosis [26]. The association between wildfire-related PM_2.5_ and cancer mortality may be explained by increased cancer incidence and shortened survival. PM_2.5_ is classified by the International Agency for Research on Cancer (IARC) as a Group 1 carcinogen for sufficient evidence in increasing lung cancer risk [27]. Assuming a potentially significant wildfire–cancer incidence association, cancer patients with weakened immune systems may be more vulnerable to wildfire-related PM_2.5_, resulting in a significant mortality association [28,29]. Although the mechanisms are not clear, some other studies have provided evidence for related cancers. According to previous studies, all-source PM_2.5_ exposure may shorten cancer survival [30,31]. The potential mechanism for shortened survival may be accelerated cancer progression due to PM_2.5_ inducing oxidative stress, genotoxicity, and/or inflammation [32–36]. PM_2.5_ may enter the digestive tract, altering the immune response and the gut microbiota and epithelial cells [37]. Moreover, PM_2.5_ binds chemicals with endocrine-disrupting properties [38] that may be associated with cancer development and progression of hormone-sensitive cancers, such as breast and testicular cancer. Findings from an epidemiological study of breast cancer [39] and mechanistic studies of testicular cancer [40] also support the hypothesis. Therefore, it is biologically plausible that exposure to wildfire-related PM_2.5_ might increase mortality for cancers at different sites.

Wildfire particles are smaller than those from urban sources, and particles reaching miles away may have greater oxidative potential [41,42]. These characteristics of wildfire particles pose a significant health risk to individuals. The health impact of short-term exposure to wildfire smoke has been well documented for all-cause mortality [1,43]. In comparison with the short-term effects of wildfire smoke, far fewer studies have included longer term health impacts, and no study to our knowledge has shown an association between wildfire particles and cancer. Some studies have reported an association between all-source PM_2.5_ and cancer risks [44]. Our results show a higher risk for wildfire-related PM_2.5_ than for PM_2.5_ from non-wildfire source, for deaths from all cancers combined. Furthermore, we might expect that cancer at different sites would show different responses to wildfire-related PM_2.5_ exposure.

In addition to the short-term effect, other effects of wildfire smoke exposure on cancer risk are still unknown in the general population, but higher cancer risks were observed among wildfire firefighters who were most exposed to wildfire smoke. The American Cancer Society (ACS) Cancer Prevention Study II demonstrated that wildland firefighters have an increased risk of lung cancer mortality [45]. Studies from Australia showed possible increased risks of colorectal and prostate cancers in paid male landscape firefighters, colorectal and kidney cancer in male firefighters, and all malignancies in female landscape firefighters [46–48]. However, no increased cancer mortality risks were observed, which may be due to the “healthy volunteer” effect [46–48]. No other studies of cancer among wildfire firefighters were identified in the literature. The risks of cancer in firefighters, not limited to wildfires, are still uncertain. In line with our results, increased risks of some respiratory system, digestive tract, skin, and male reproductive cancers were reported in firefighters, but some protective associations were also observed, which again may be due to the healthy volunteer effect [49–54]. Consistent with previous studies, lung cancer was not observed to have a higher mortality risk associated with wildfire-related PM_2.5_ exposure, while statistically significant associations were observed in female and older populations with non-wildfire PM_2.5_ in the current study [46–54]. However, our previous study conducted in the same population and over a similar study period showed significant associations between all-source PM_2.5_ and lung cancer mortality for all subgroups (both sexes and age groups) [55]. The increased risk of lung cancer mortality may be explained by non-wildfire sources of PM_2.5_ exposure, such as industry and transportation emissions, but not wildfires. Further studies on different sources of PM_2.5_ and histological subtype-specific estimation are warranted.

Another kind of exposure similar to wildfire smoke is the emission from biomass burning. The literature suggests that indoor biomass burning is associated with lung cancer risk [56,57]. Robust evidence has been provided that biomass smoke from household cooking and heating is also associated with higher risks of gastrointestinal cancers [58–60]. Furthermore, indoor wood-burning stoves were suggested to be associated with a modestly increased risk of breast cancer in the Sister Study from the US [61]. An increased risk of hypopharyngeal cancer was also observed among lifelong users of wood in a case–control study from India [62]. Most studies were not able to assess the effect of biomass burning separately, as participants using fossil fuels included biomass and coal as cooking and heating fuels. However, in vitro studies have demonstrated that particles emitted by wood fires have mutagenic and endocrine-disrupting capacity, providing biological support for the possible role of wildfires in the pathogenesis of hormone-sensitive cancers [63,64].

Although both firefighter smoke exposure and indoor biomass burning pollution may not be directly comparable with wildfire-related PM_2.5_ exposure in the general population, this literature sheds some light on cancer-specific risks. The current analysis establishes a higher risk for cancer, which means better control of wildfires is essential for cancer prevention in Brazil. In addition, individual-, community-, and national-level strategies should be considered to minimize fire exposure. However, the effectiveness of personal actions such as relocation, staying indoors, or wearing masks is still controversial [2]. The investigation of wildfire prediction models applied to Brazil is ongoing [65]. Warning systems for extremely hot weather implemented in many countries may also provide a warning for increased wildfire risk [66]. Systemic strategies and guidance are warranted.

Major strengths of this study include that it is the first study to estimate the association between wildfire-related PM_2.5_ and cancer-specific mortality, to our best knowledge. Also, this study is based on national death records, and the large sample size allowed the estimation of associations representative for the total Brazilian population. Lastly, the variant difference-in-differences approach could adjust for most of the unmeasured confounders stable during the study time and those that changed similarly across regions.

Some study limitations should also be recognized. First, we considered only PM_2.5_ exposure in the analyses, and potential joint effects of gaseous pollutants were not estimated. Our estimation of wildfire-related PM_2.5_ could not capture the complex mixture of environmental pollutants released during wildfires, and thus further studies are needed to refine the exposure metrics. Additionally, some confounders related to wildfires could not be adjusted for in the model due to data unavailability, including data on firefighting foam composition. For example, associations between firefighting chemicals and cancer at various cancer sites have been observed in previous studies: Firefighting foam containing per- and polyfluoroalkyl substances was suggested to be related to increased risk of breast cancer, bladder cancer, etc. [67]. Second, PM_2.5_ concentrations were estimated at a global scale, and regional validation was not available due to limited ground-level monitors in Brazil. Third, municipality-level exposures were used in the analyses because individual exposure data were not available. The use of aggregated data may lead to some exposure misclassification, including the inability to capture the migration of residents between municipalities. However, potential effects of migration may be limited, as more than 96% of adults had an uninterrupted time of residence in a municipality for at least 2 years according, according to the 2010 census results published by BIGS [68]. Finally, the use of registry data, rather than individual survey data, may have led to some misclassification of residential address and main cause of death, which may lead to bias in the association estimations. Some potentially changing confounders that correlated with both PM_2.5_ exposure and cancer mortality were ignored in the analyses, due to the limited availability of individual lifestyle data. Further, the data used in this study did not allow an assessment of competing risks, as only the primary cause of death was recorded. Cancer patients who died from other causes (e.g., heart attack) could not be included. Also, our use of 2-year average PM_2.5_ concentration as the exposure may be not appropriate for cancers with a longer survival time. Overall, further cohort studies are warranted to give a more accurate risk estimate.

In summary, this study provides the first quantitative estimate of the association between wildfire-related PM_2.5_ and cancer-specific mortality across Brazil. The potentially higher risk of wildfire-related PM_2.5_ compared with non-wildfire-related PM_2.5_ for all cancers combined suggests that the wildfire control and systemic prevention strategies are warranted to reduce cancer mortality risk in Brazil. This could be a health co-benefit of measures to preserve the Amazon rainforest and limit climate change.

## Supporting information

S1 RECORD Checklist The RECORD statement checklist of items, extended from the STROBE statement, that should be reported in observational studies using routinely collected health data. (DOCX)

S1 Data Example data for analysis. (CSV)

S1 Fig The proportion of wildfire-related PM_2.5_ of all-source PM_2.5_ concentration during 2010–2016. The base map of this figure was downloaded from the Brazilian Institute of Geography and Statistics (https://www.ibge.gov.br/en/geosciences/territorial-organization/territorial-meshes/18890-municipal-mesh.html?edicao=30154&t=downloads); the base map was free and open-access.
(TIF)

S2 Fig Annual concentration of source-specific PM_2.5_ in Brazil for the year 2017. The base map of this figure was downloaded from the Brazilian Institute of Geography and Statistics (https://www.ibge.gov.br/en/geosciences/territorial-organization/territorial-meshes/18890-municipal-mesh.html?edicao=30154&t=downloads); the base map was free and open-access. Gridded fractional source contribution results in Brazil were extracted from [69].
(TIF)

S3 Fig Estimated RRs (95% CIs) for the association between a 1-μg/m^3^ increase in single lag0−2 and moving average wildfire-related PM_2.5_ exposure and cancer mortality from 2010–2016. The horizontal dashed line represents the reference line for RR = 1, helping to compare the effect estimates with the null hypothesis; the vertical error bars represent 95% CIs. The model, by its design, controlled for factors that were stable across the study period or had similar trend across geographical locations, and also adjusted for spatial-temporal factors including seasonal temperature and GDP per capita. CI, confidence interval; RR, relative risk.
(TIF)

S4 Fig Estimated response relationship between wildfire-related PM_2.5_ and total cancer mortality, modeled by natural cubic splines with 1−4 degrees of freedom. The solid lines represent the RR, and the shaded areas represent the 95% CI. The model, by its design, controlled for factors that were stable across the study period or had similar trend across geographical locations, and also adjusted for spatial-temporal factors including seasonal temperature and GDP per capita. CI, confidence interval; RR, relative risk.
(TIF)

S5 Fig Estimated RRs and 95% CIs for the association between a 1-μg/m^3^ increase in 2-year average (lag0−1) wildfire-related PM_2.5_ and non-wildfire-related PM_2.5_ and mortality from all cancers and site-specific cancers from 2010–2016. The vertical dashed line represents the reference line for RR = 1, helping to compare the effect estimates with the null hypothesis; the error bars represent 95% CIs. The model, by its design, controlled for factors that were stable across the study period or had similar trend across geographical locations, and also adjusted for spatial-temporal factors including seasonal temperature and GDP per capita. *p*-Values for differences were estimated by fixed-effects meta-analysis with no statistical adjustment, because models were based on the same sample. CI, confidence interval; RR, relative risk.
(TIF)

S6 Fig Estimated RRs (95% CIs) for the associations between a 1-μg/m^3^ increase in 2-year average (lag0–1) wildfire-related PM_2.5_ and mortality from all cancers and site-specific cancers from 2010–2016, by sex and age. The horizontal dashed line represents the reference line for RR = 1, helping to compare the effect estimates with the null hypothesis; the vertical error bars represent 95% CIs. The model, by its design, controlled for factors that were stable across the study period or had similar trend across geographical locations, and also adjusted for spatial-temporal factors including seasonal temperature and GDP per capita. CI, confidence interval; RR, relative risk.
(TIF)

S7 Fig Comparison of the estimated RRs (95% CIs) for the association between a 1-μg/m^3^ increase in 2-year average (lag0−1) wildfire- and non-wildfire-related PM_2.5_ and lung cancer mortality from 2010–2016, by sex and age. The horizontal dashed line represents the reference line for RR = 1, helping to compare the effect estimates with the null hypothesis; the vertical error bars represent 95% CIs. The model, by its design, controlled for factors that were stable across the study period or had similar trend across geographical locations, and also adjusted for spatial-temporal factors including seasonal temperature and GDP per capita. CI, confidence interval; RR, relative risk.
(TIF)

S1 Table Cancer death counts for common cancer sites by age and sex during 2010–2016 in Brazil. (DOCX)

S2 Table Cancer deaths and attributable cancer deaths associated with the 2-year average wildfire-related PM_2.5_ exposure of each state in Brazil during 2010–2016. (DOCX)

S3 Table Results of sensitivity analyses changing covariates and degrees of freedom of temperature for total cancer deaths. (DOCX)

S4 Table Results of sensitivity analyses for total cancer and negative control mortality. (DOCX)

S1 Text R code for analysis. (DOCX)

## Abbreviations

BIGS: Brazilian Institute of Geography and Statistics
GDP: gross domestic product
NDVI: Normalized Difference Vegetation Index
RR: relative risk
