# Supplementary figures and images for: Exposure to wildfire-related PM2.5 and site-specific cancer mortality in Brazil from 2010 to 2016: A retrospective study
Source: PLoS Med. 2022 Sep 19;19(9):e1004103. doi: 10.1371/journal.pmed.1004103 (PMC9529133; doi:10.1371/journal.pmed.1004103)

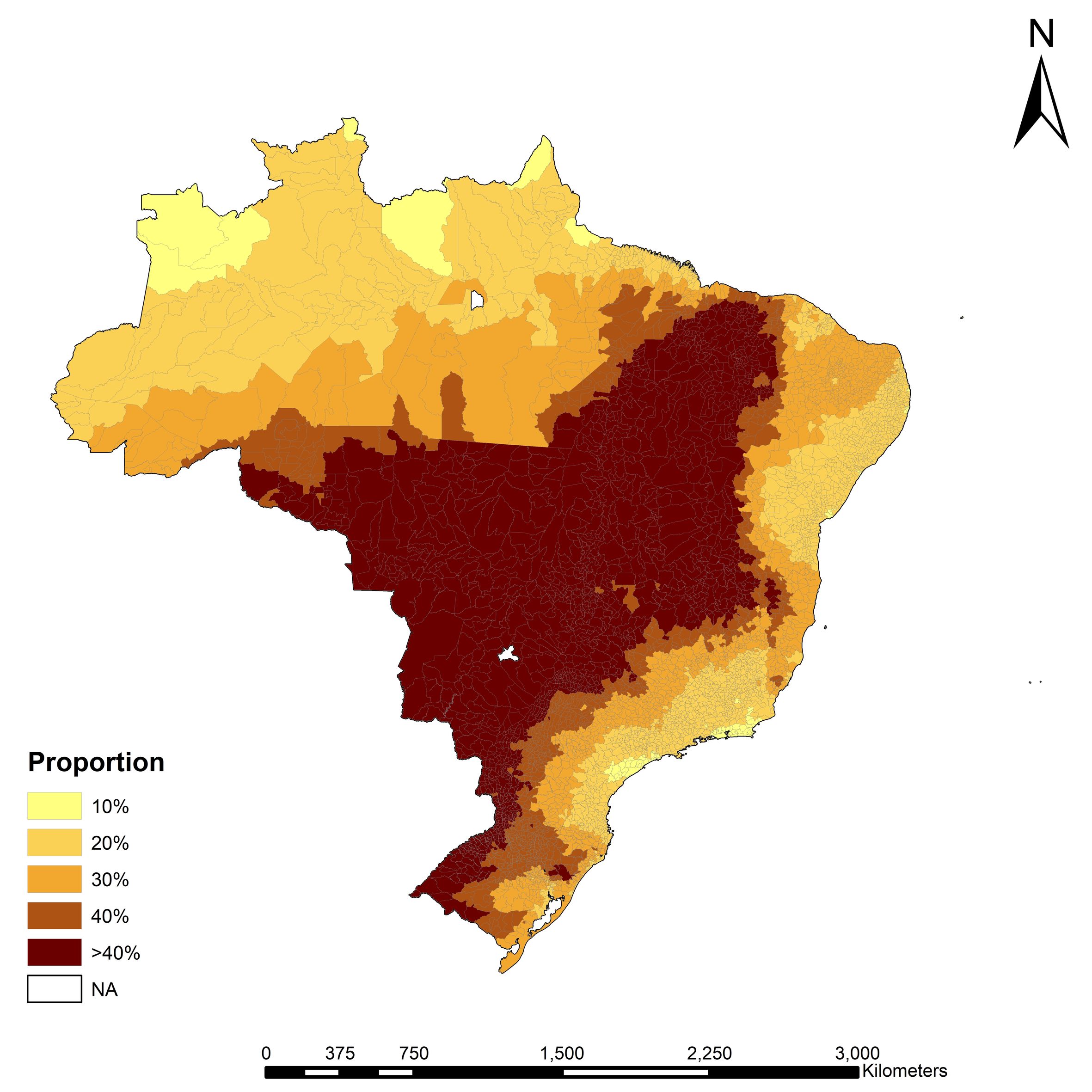

Supplement: S1 Fig — The base map of this figure was downloaded from the Brazilian Institute of Geography and Statistics (https://www.ibge.gov.br/en/geosciences/territorial-organization/territorial-meshes/18890-municipal-mesh.html?edicao=30154&t=downloads); the base map was free and open-access. (TIF) [file pmed.1004103.s003.tif]

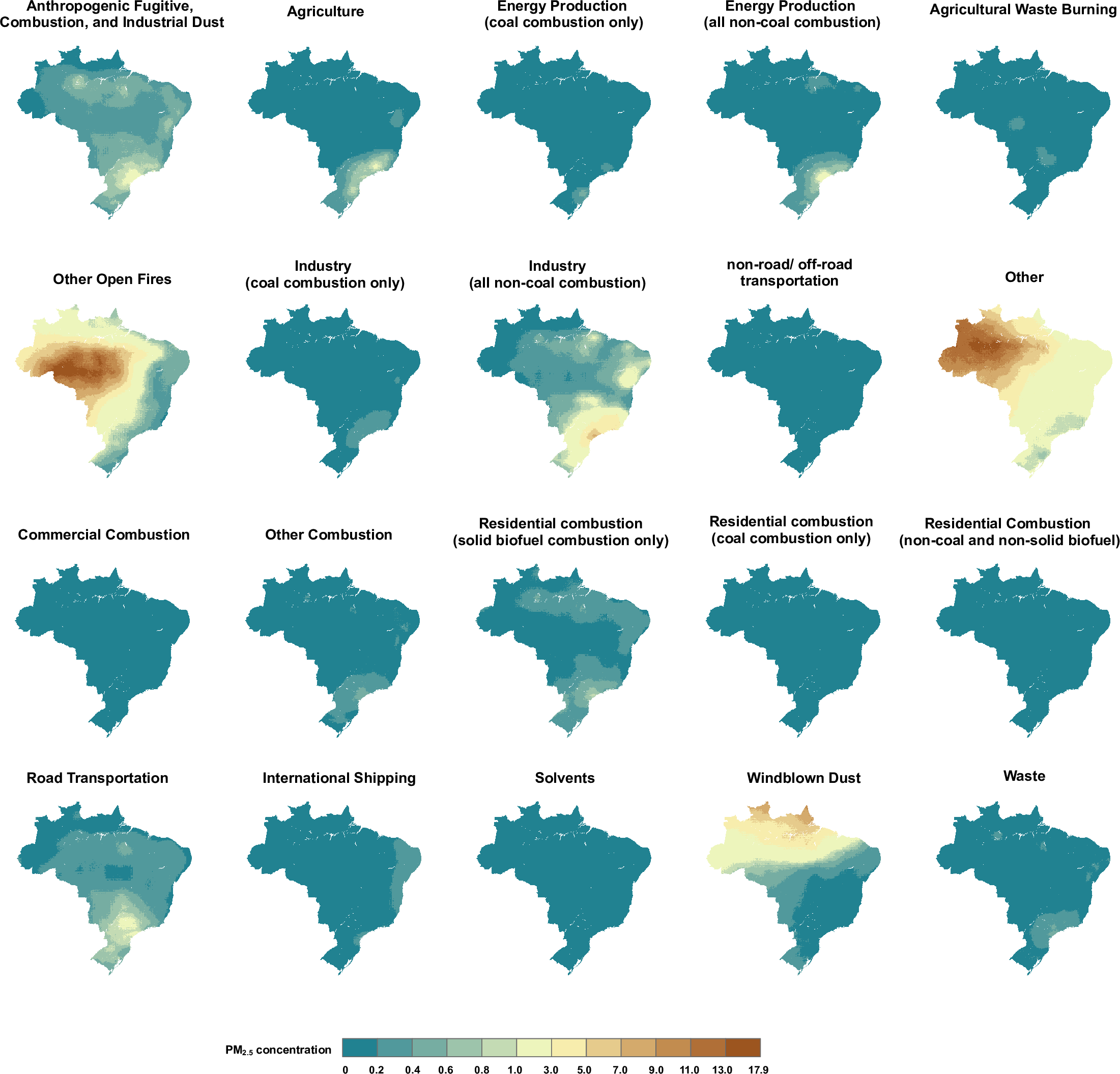

Supplement: S2 Fig — The base map of this figure was downloaded from the Brazilian Institute of Geography and Statistics (https://www.ibge.gov.br/en/geosciences/territorial-organization/territorial-meshes/18890-municipal-mesh.html?edicao=30154&t=downloads); the base map was free and open-access. Gridded fractional source contribution results in Brazil were extracted from [69]. (TIF) [file pmed.1004103.s004.tif]

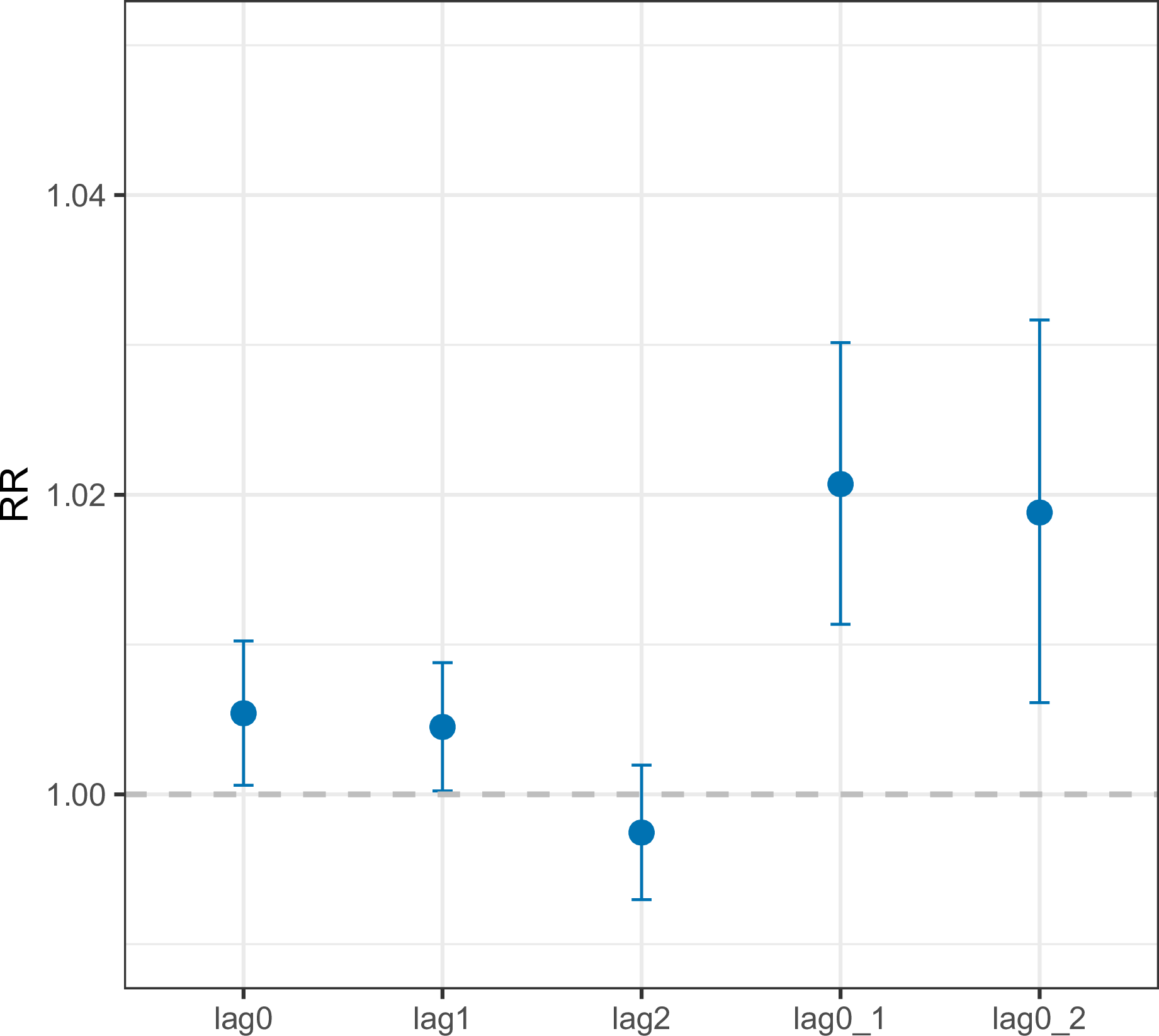

Supplement: S3 Fig — The horizontal dashed line represents the reference line for RR = 1, helping to compare the effect estimates with the null hypothesis; the vertical error bars represent 95% CIs. The model, by its design, controlled for factors that were stable across the study period or had similar trend across geographical locations, and also adjusted for spatial-temporal factors including seasonal temperature and GDP per capita. CI, confidence interval; RR, relative risk. (TIF) [file pmed.1004103.s005.tif]

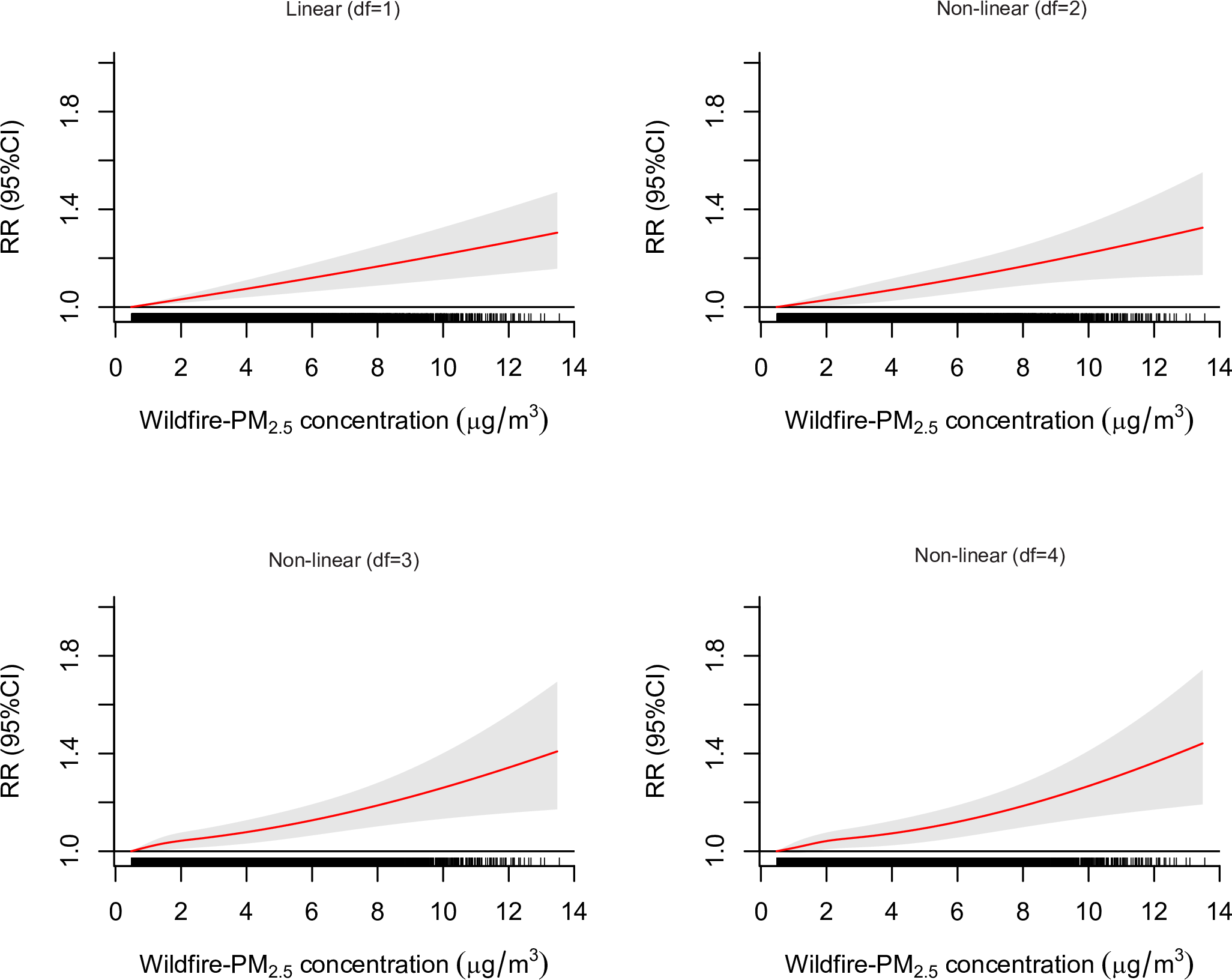

Supplement: S4 Fig — The solid lines represent the RR, and the shaded areas represent the 95% CI. The model, by its design, controlled for factors that were stable across the study period or had similar trend across geographical locations, and also adjusted for spatial-temporal factors including seasonal temperature and GDP per capita. CI, confidence interval; RR, relative risk. (TIF) [file pmed.1004103.s006.tif]

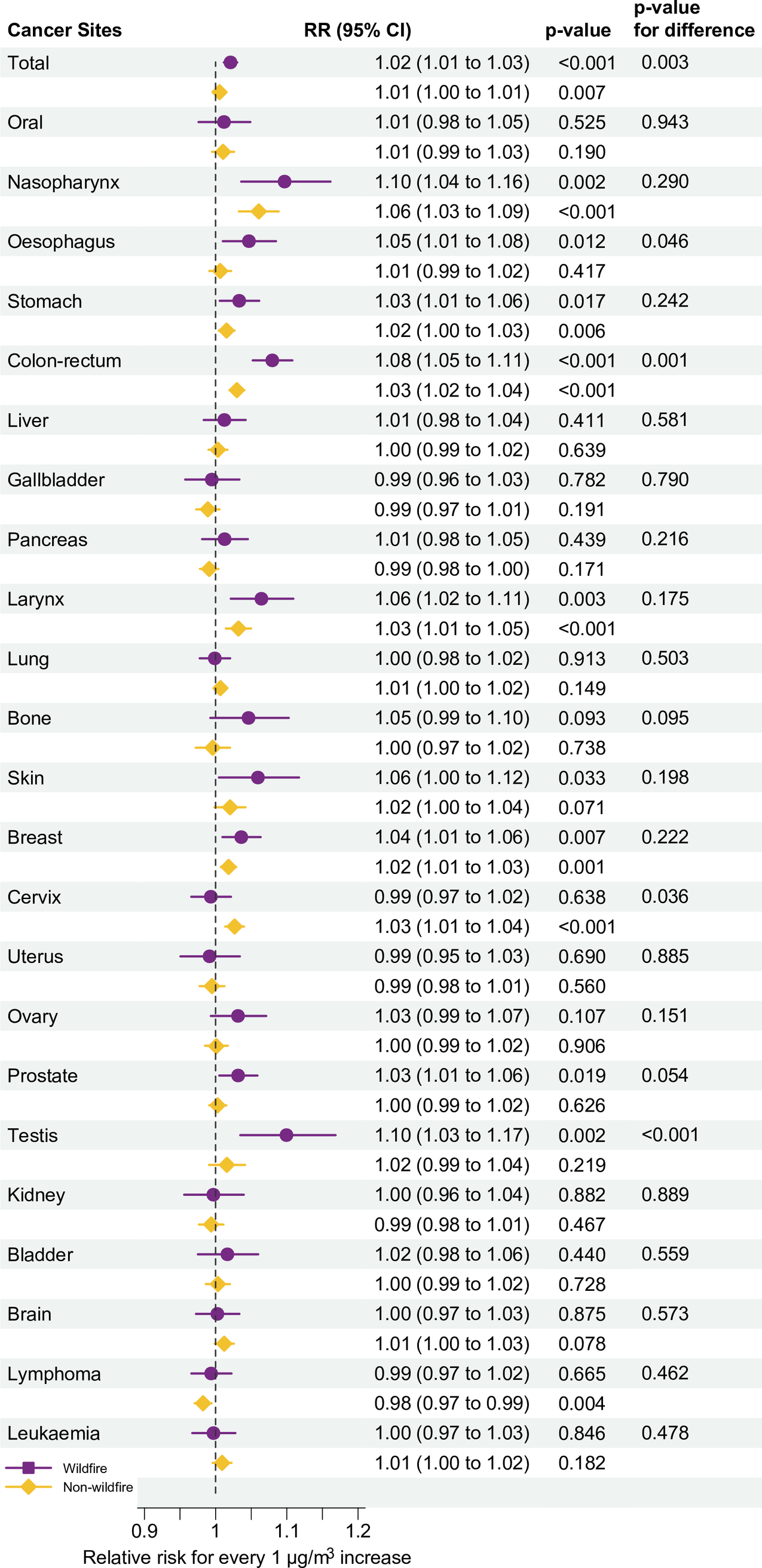

Supplement: S5 Fig — The vertical dashed line represents the reference line for RR = 1, helping to compare the effect estimates with the null hypothesis; the error bars represent 95% CIs. The model, by its design, controlled for factors that were stable across the study period or had similar trend across geographical locations, and also adjusted for spatial-temporal factors including seasonal temperature and GDP per capita. p-Values for differences were estimated by fixed-effects meta-analysis with no statistical adjustment, because models were based on the same sample. CI, confidence interval; RR, relative risk. (TIF) [file pmed.1004103.s007.tif]

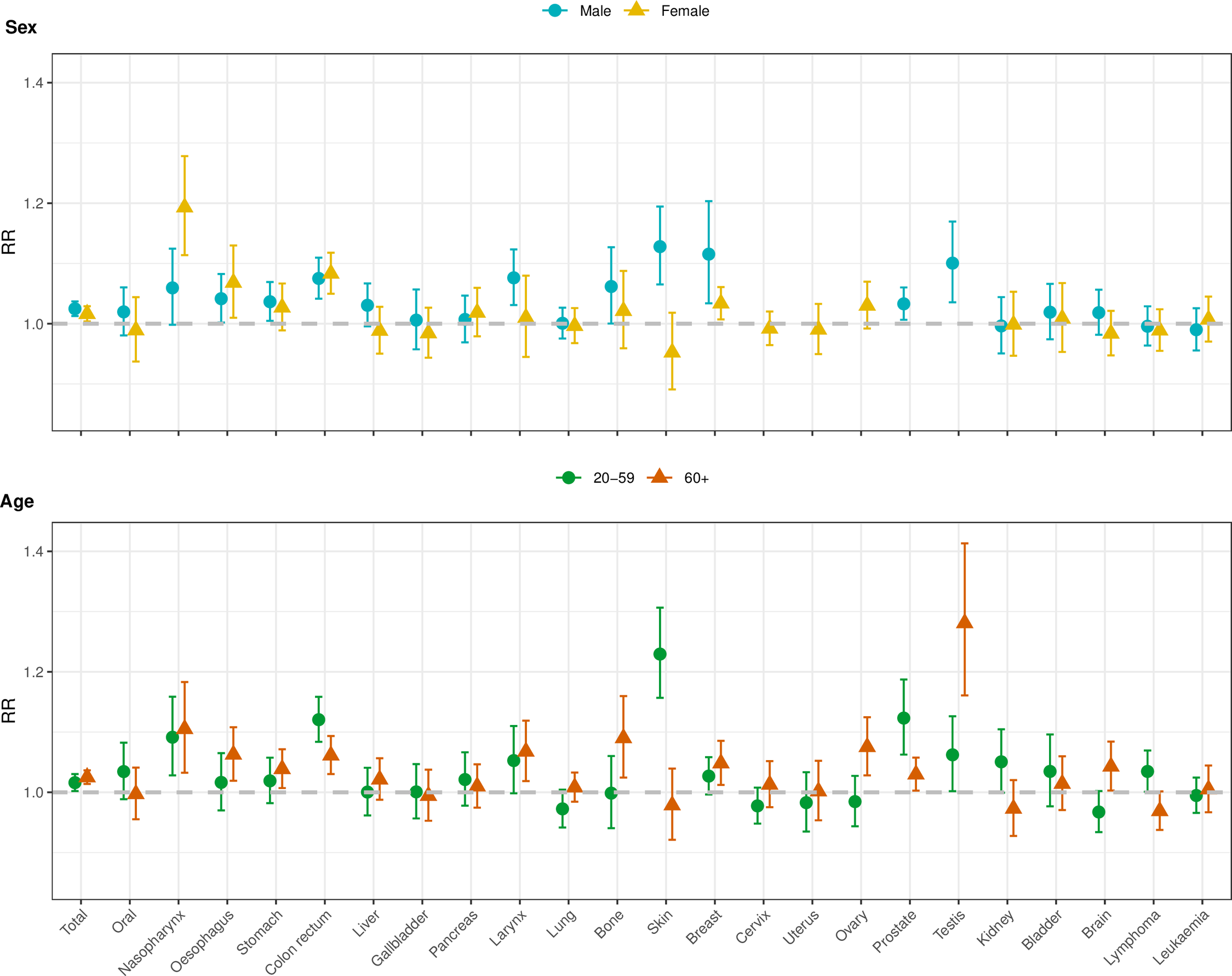

Supplement: S6 Fig — The horizontal dashed line represents the reference line for RR = 1, helping to compare the effect estimates with the null hypothesis; the vertical error bars represent 95% CIs. The model, by its design, controlled for factors that were stable across the study period or had similar trend across geographical locations, and also adjusted for spatial-temporal factors including seasonal temperature and GDP per capita. CI, confidence interval; RR, relative risk. (TIF) [file pmed.1004103.s008.tif]

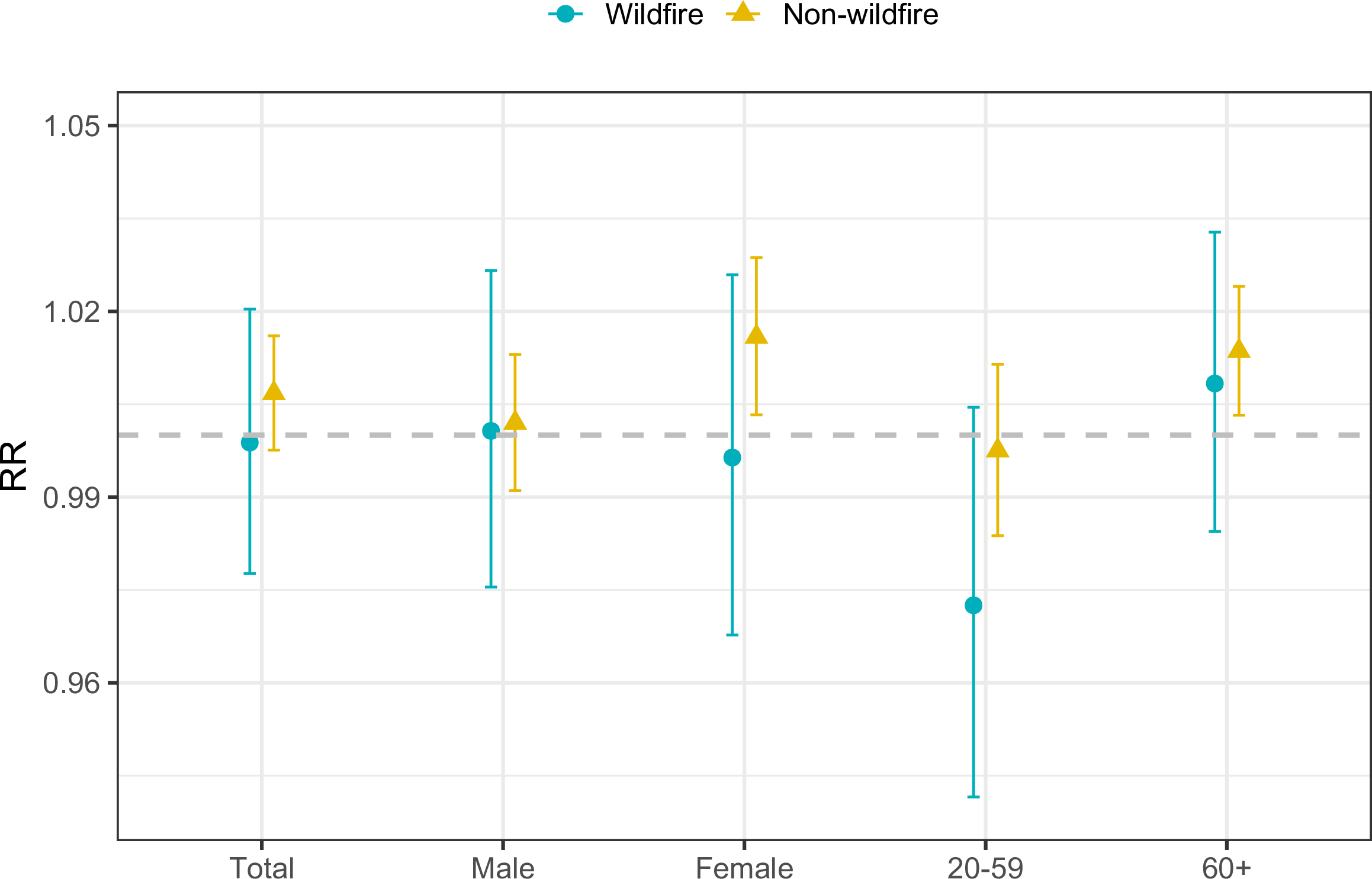

Supplement: S7 Fig — The horizontal dashed line represents the reference line for RR = 1, helping to compare the effect estimates with the null hypothesis; the vertical error bars represent 95% CIs. The model, by its design, controlled for factors that were stable across the study period or had similar trend across geographical locations, and also adjusted for spatial-temporal factors including seasonal temperature and GDP per capita. CI, confidence interval; RR, relative risk. (TIF) [file pmed.1004103.s009.tif]
