# Supplementary material for: Exposure to wildfire-related PM2.5 and site-specific cancer mortality in Brazil from 2010 to 2016: A retrospective study
Source: PLoS Med. 2022 Sep 19;19(9):e1004103. doi: 10.1371/journal.pmed.1004103 (PMC9529133; doi:10.1371/journal.pmed.1004103)
Supplement: S1 Table — (DOCX) [file pmed.1004103.s010.docx]

**S1 Table**. Cancer death counts from common cancer sites by age and sex during 2010-2016 in Brazil.

| **Cancer sites** | **All** | **Males (%)** | **Females (%)** | **20-59 y (%)** | **60+ y (%)** |
| --- | --- | --- | --- | --- | --- |
| Total | 1,332,526 | 709,535 (53.25) | 622,887 (46.74) | 420,792 (31.58) | 911,734 (68.42) |
| Oral | 48,601 | 38,712 (79.65) | 9,887 (20.34) | 20,817 (42.83) | 27,784 (57.17) |
| Nasopharynx | 2,051 | 1,414 (68.94) | 637 (31.06) | 1,130 (55.10) | 921 (44.90) |
| Oesophagus | 55,734 | 43,317 (77.72) | 12,414 (22.27) | 20,319 (36.46) | 35,415 (63.54) |
| Stomach | 97,409 | 62,578 (64.24) | 34,826 (35.75) | 28,509 (29.27) | 68,900 (70.73) |
| Colon-rectum | 107,942 | 52,411 (48.55) | 55,516 (51.43) | 31,178 (28.88) | 76,764 (71.12) |
| Liver | 61,646 | 35,312 (57.28) | 26,327 (42.71) | 17,944 (29.11) | 43,702 (70.89) |
| Gallbladder | 21,643 | 7,446 (34.40) | 14,196 (65.59) | 5,381 (24.86) | 16,262 (75.14) |
| Pancreas | 60,301 | 29,965 (49.69) | 30,327 (50.29) | 14,865 (24.65) | 45,436 (75.35) |
| Larynx | 28,849 | 25,220 (87.42) | 3,624 (12.56) | 10,949 (37.95) | 17,900 (62.05) |
| Lung | 171,089 | 10,2816 (60.10) | 68,255 (39.89) | 43,384 (25.36) | 127,705 (74.64) |
| Bone | 10,700 | 6,237 (58.29) | 4,462 (41.70) | 4,595 (42.94) | 6,105 (57.06) |
| Skin | 11,163 | 6,414 (57.46) | 4,749 (42.54) | 4,239 (37.97) | 6,924 (62.03) |
| Breast | 100,889 | 1,135 (1.12) | 99,745 (98.87) | 48,642 (48.21) | 52,247 (51.79) |
| Cervix | 37,815 | - | 37,815 (100) | 21,860 (57.81) | 15,955 (42.19) |
| Uterus | 23,957 | - | 23,957 (100) | 8,357 (34.88) | 15,600 (65.12) |
| Ovary | 22,877 | - | 22,877 (100) | 9,472 (41.40) | 13,405 (58.60) |
| Prostate | 96,501 | 96,501 (100) | - | 4,846 (5.02) | 91,655 (94.98) |
| Testis | 2,054 | 2,054 (100) | - | 1,764 (85.88) | 290 (14.12) |
| Kidney | 21,018 | 13,135 (62.49) | 7,881 (37.50) | 6,274 (29.85) | 14,744 (70.15) |
| Bladder | 25,019 | 17,236 (68.89) | 7,780 (31.10) | 3,293 (13.16) | 21,726 (86.84) |
| Brain | 54,326 | 28,246 (51.99) | 26,078 (48.00) | 24,855 (45.75) | 29,471 (54.25) |
| Lymphoma | 50,261 | 26,822 (53.37) | 23,433 (46.62) | 17,019 (33.86) | 33,242 (66.14) |
| Leukaemia | 38,613 | 20,548 (53.22) | 18,065 (46.78) | 15,140 (39.21) | 23,473 (60.79) |
