# Supplementary material for: Exposure to wildfire-related PM2.5 and site-specific cancer mortality in Brazil from 2010 to 2016: A retrospective study
Source: PLoS Med. 2022 Sep 19;19(9):e1004103. doi: 10.1371/journal.pmed.1004103 (PMC9529133; doi:10.1371/journal.pmed.1004103)
Supplement: S2 Table — (DOCX) [file pmed.1004103.s011.docx]

**S2 Table**. Cancer deaths and attributable cancer deaths associated with 2-year average wildfire related PM_2.5_ exposure of each State in Brazil during 2010-2016

| **State** | **Wildfire related PM_2.5_** | **Cancer deaths** | **Cancer mortality (/10^6^)** | **Attributable cancer deaths** | **Attributable cancer deaths (/10^6^)** |
| --- | --- | --- | --- | --- | --- |
| **Central-west** |  |  |  |  |  |
| Federal district | 32.72 | 18,976 | 999.78 | 1,114 (621−1,603) | 58.71 (32.72−84.46) |
| Goiás | 30.45 | 33,367 | 760.96 | 2,396 (1,335-3,446) | 54.63 (30.45-78.6) |
| Mato Grosso | 60.77 | 15,058 | 705.59 | 2,327 (1,297-3,348) | 109.04 (60.77-156.87) |
| Mato Grosso do Sul | 44.74 | 15,746 | 908.26 | 1,392 (776-2,002) | 80.27 (44.74-115.48) |
| **North** |  |  |  |  |  |
| Acre | 29.79 | 3,000 | 665.81 | 241 (134-347) | 53.46 (29.79-76.91) |
| Amapá | 7.35 | 2,175 | 512.62 | 56 (31-80) | 13.18 (7.35-18.96) |
| Amazonas | 20.33 | 15,062 | 695.38 | 790 (440-1,137) | 36.48 (20.33-52.49) |
| Pará | 12.00 | 25,849 | 535.09 | 1,040 (580-1,496) | 21.53 (12-30.97) |
| Rondônia | 42.93 | 6,531 | 611.22 | 823 (459-1,184) | 77.03 (42.93-110.82) |
| Roraima | 11.11 | 1,566 | 548.47 | 57 (32-82) | 19.94 (11.11-28.69) |
| Tocantins | 44.52 | 6,292 | 683.46 | 735 (410-1,058) | 79.89 (44.52-114.93) |
| **Northeast** |  |  |  |  |  |
| Alagoas | 5.77 | 12,710 | 630.34 | 209 (116-300) | 10.35 (5.77-14.89) |
| Bahia | 7.19 | 69,004 | 716.2 | 1,243 (693-1,788) | 12.9 (7.19-18.55) |
| Ceará | 10.50 | 51,990 | 896.92 | 1,092 (609-1,571) | 18.84 (10.5-27.11) |
| Maranhão | 14.97 | 21,855 | 530.88 | 1,106 (616-1,591) | 26.86 (14.97-38.64) |
| Paraíba | 8.29 | 23,138 | 888.81 | 387 (216-557) | 14.87 (8.29-21.4) |
| Pernambuco | 7.92 | 54,195 | 892.64 | 863 (481-1,241) | 14.21 (7.92-20.44) |
| Piauí | 30.83 | 17,178 | 822.36 | 1,155 (644-1,662) | 55.31 (30.83-79.57) |
| Rio Grande do Norte | 8.90 | 20,031 | 894.65 | 358 (199-515) | 15.98 (8.9-22.98) |
| Sergipe | 6.07 | 10,988 | 778.17 | 154 (86-221) | 10.89 (6.07-15.67) |
| **South** |  |  |  |  |  |
| Paraná | 32.36 | 88,429 | 1,165.18 | 4,406 (2,456-6,339) | 58.06 (32.36-83.52) |
| Rio Grande do Sul | 29.62 | 120,788 | 1,514.93 | 4,237 (2,361-6,095) | 53.14 (29.62-76.45) |
| Santa Catarina | 20.35 | 51,915 | 1,105 | 1,715 (956-2,467) | 36.51 (20.35-52.52) |
| **Southeast** |  |  |  |  |  |
| Espírito Santo | 11.39 | 26,101 | 1,009.77 | 528 (294-760) | 20.43 (11.39-29.39) |
| Minas Gerais | 20.21 | 135,570 | 945.49 | 5,200 (2,898-7,481) | 36.27 (20.21-52.18) |
| Rio de Janeiro | 12.92 | 140,470 | 1,177.66 | 2,765 (1,541-3,978) | 23.18 (12.92-33.35) |
| São Paulo | 25.65 | 344,542 | 1,114.45 | 14,231 (7,931-20,473) | 46.03 (25.65-66.22) |
