# Supplementary material for: Exposure to wildfire-related PM2.5 and site-specific cancer mortality in Brazil from 2010 to 2016: A retrospective study
Source: PLoS Med. 2022 Sep 19;19(9):e1004103. doi: 10.1371/journal.pmed.1004103 (PMC9529133; doi:10.1371/journal.pmed.1004103)
Supplement: S3 Table — (DOCX) [file pmed.1004103.s012.docx]

**S3 Table.** Results of sensitivity analyses changing covariates and df of temperature for total cancers.

| **Model** | **RR (95% CI)** | ***p-value*** | ***p*-value for difference** |
| --- | --- | --- | --- |
| Primary | 1.021 (1.012−1.030) | <.001 | Ref |
| Adjusting for CO | 1.022 (1.013-1.032) | <.001 | 0.821 |
| Adjusting for NO_2_ | 1.022 (1.012-1.031) | <.001 | 0.915 |
| Adjusting for O_3_ | 1.021 (1.012-1.031) | <.001 | 0.956 |
| Adjusting for SO_2_ | 1.019 (1.009-1.029) | <.001 | 0.775 |
| Adjusting for max NTL | 1.021 (1.012-1.030) | <.001 | 0.993 |
| Adjusting for NDVI | 1.019 (1.010-1.029) | <.001 | 0.803 |
| Adjusting for CO+ NO_2_+O_3_+ SO_2_+NTL+NDVI | 1.020 (1.009-1.031) | <.001 | 0.908 |
| Adjusting for hot and cold season temperature | 1.022 (1.013-1.032) | <.001 | 0.821 |
| Not adjusting for non-wildfire PM_2.5_ | 1.021 (1.011-1.030) | <.001 | 0.976 |
| Not adjusting for GDP per capita | 1.021 (1.012-1.030) | <.001 | 0.986 |
| Not adjusting for temperature | 1.014 (1.005-1.022) | 0.002 | 0.261 |
| df of temperature = 2 | 1.019 (1.009-1.028) | <.001 | 0.750 |
| df of temperature = 3 | 1.018 (1.009-1.028) | <.001 | 0.693 |

Note: P-value for difference were estimated by fix effect meta-analysis with no statistical adjustment, because those models were based on the same sample. NDVI: Normalized Difference Vegetation Index; NTL: Nighttime Light; Annual CO (p.p.b.), NO_2_ (p.p.b.), and O_3_ (p.p.b.) concentrations were obtained from the Environmental Information System for Health (http://queimadas.dgi.inpe.br/queimadas/sisam/v2/dados/download/). NDVI data was extracted from the product of MOD13Q1.006 (<https://lpdaac.usgs.gov/products/mod13q1v006/>). NTL data was extracted from an extended time-series (2000-2018) of global NPP-VIIRS-like nighttime light data (https://dataverse.harvard.edu/dataset.xhtml?persistentId=doi:10.7910/DVN/YGIVCD). Df,degree of freedom; RR, relative risk; CI, confidence interval.
