# Supplementary material for: Exposure to wildfire-related PM2.5 and site-specific cancer mortality in Brazil from 2010 to 2016: A retrospective study
Source: PLoS Med. 2022 Sep 19;19(9):e1004103. doi: 10.1371/journal.pmed.1004103 (PMC9529133; doi:10.1371/journal.pmed.1004103)
Supplement: S4 Table — (DOCX) [file pmed.1004103.s013.docx]

**S4 Table**. Results of sensitivity analyses for total cancers and negative control.

| Outcome | RR (95% CI) | *p-value* |
| --- | --- | --- |
| Cancer | 1.021 (1.012−1.030) | <.001 |
| Drowning | 1.046 (0.983-1.114) | 0.158 |

Note: RR, relative risk; CI, confidence interval.
