# Supplementary material for: Exposure to wildfire-related PM2.5 and site-specific cancer mortality in Brazil from 2010 to 2016: A retrospective study
Source: PLoS Med. 2022 Sep 19;19(9):e1004103. doi: 10.1371/journal.pmed.1004103 (PMC9529133; doi:10.1371/journal.pmed.1004103)
Supplement: S1 Text — (DOCX) [file pmed.1004103.s014.docx]

##R codes for difference in differences ##
#### Be aware that this is simulated data. It is not the same as the study data

##Load example data

data<- read.csv("data_sample.csv")

##Start to perform the analysis

library(gnm)

##Take total death as an example

model<-gnm(Death~PM+summer_mean+winter_mean+summer_sd+winter_sd+GDP+log(Population)+as.factor(year),

family = quasipoisson,eliminate = as.factor(citycode),data=data)

summary(model)
